# Characterization of grass carp reovirus minor core protein VP4

**DOI:** 10.1186/1743-422X-9-89

**Published:** 2012-07-05

**Authors:** Liming Yan, Hong Guo, Xiaoyun Sun, Ling Shao, Qin Fang

**Affiliations:** 1 State Key Laboratory of Virology, Wuhan Institute of Virology, Chinese Academy of Sciences, Wuhan, 430071, China; 2 Graduate School of the Chinese Academy of Sciences, Beijing, 100039, China

## Abstract

**Background:**

Grass Carp Reovirus (GCRV), a tentative member in the genus *Aquareovirus* of family *Reoviridae*, contains eleven segmented (double-stranded RNA**)** dsRNA genome which encodes 12 proteins. A low-copy core component protein VP4, encoded by the viral genome segment 5(S5), has been suggested to play a key role in viral genome transcription and replication.

**Results:**

To understand the role of minor core protein VP4 played in molecular pathogenesis during GCRV infection, the recombinant GCRV VP4 gene was constructed and expressed in both prokaryotic and mammalian cells in this investigation. The recombinant His-tag fusion VP4 products expressed in E.*coli* were identified by Western blotting utilizing His-tag specific monoclonal and GCRV polyclonal antibodies. In addition, the expression of VP4 in GCRV infected cells, appeared in granules structure concentrated mainly in the cytoplasm, can be detected by Immunofluorescence (IF) using prepared anti-VP4 polyclonal antibody. Meanwhile, VP4 protein in GCRV core and infected cell lysate was identified by Immunoblotting (IB) assay. Of particular note, the VP4 protein was exhibited a diffuse distribution in the cytoplasm and nucleus in transfected cells, suggesting that VP4 protein may play a partial role in the nucleus by regulating cell cycle besides its predicted cytoplasmic function in GCRV infection.

**Conclusions:**

Our results indicate the VP4 is a core component in GCRV. The cellular localization of VP4 is correlated with its predicted function. The data provide a foundation for further studies aimed at understanding the role of VP4 in viroplasmic inclusion bodies (VIB) formation during GCRV replication and assembly.

## Background

Double stranded (ds) RNA viruses, which affect a very wide range of host species including vertebrates, invertebrates, plants, fungi, and prokaryotes, represent a diverse group of viral pathogens [1]. According to classification of the International Committee on Taxonomy of Viruses (ICTV), eight distinct families are currently recognized [2]. The *Reoviridae*, one of the most complex families in dsRNA group, consists of at least 15 distinct genera reported so far. The virus particles in the family of *Reoviridae* appear to be icosahedral in symmetry with an overall diameter of approximately 60–85 nm comprising 9–12 segmented dsRNA genome enclosed within multiple concentric protein capsids. Based on their structure organization, it is possible to divide the members of the family *Reoviridae* into two subfamilies, *Spinareovirinae* and *Sedoreovirinae*[2]. The members of the *Spinareovirinae* subfamily have 12 icosahedrally pentameric turrets resided on the surface of core or at the fivefold axe of intact particle (eg. orthoreoviruses or cypoviruses), while the viruses in the *Sedoreovirinae* subfamily possess a relatively smooth surface and core without large surface projections at their fivefold axes (eg. rotaviruses or orbiviruses). GCRV (grass carp reovirus), a tentative member of genus *Aquareovirus*, could be classified into *Spinareovirinae* subfamily of *Reoviridae*[2].

GCRV has been recognized as the most pathogenic amongst all the isolated aquareoviruses [3,4]. Similar to other members of *Reoviridae*, GCRV is a multilayer spherical particle enclosing a dsRNA genome of 11 segments, which encode 7 structural proteins (VP1-VP7) and 5 nonstructural proteins. Amongst the 7 structural proteins, VP1-VP4 and VP6 proteins are the components of viral core, and the outer capsid of GCRV is made up of 200 trimers of VP5-VP7 heterodimers organized into an incomplete T = 13 lattice. The core is a T = 1 particle with 12 VP1 pentameric turrets decorating a shell of 60 VP3 dimers, which are clamped together by 120 VP6 monomers [5-8], while the other two core proteins, VP2 and VP4, appeared in a low copy located near the VP1 turret protein. The structure and function of the viral major constitute proteins have been well resolved according to recent progress on GCRV atom image [8], however, the minor structural proteins VP2 and VP4 that are related to enzyme activity in RNA transcription and replication are poorly understood due to their low copy in mature viral particles.

Previous study of genome sequences of aquareoviruses indicated that aquareoviruses have a common evolutionary origin with genus *Orthoreovirus*, including mammalian reoviruses(MRV) and avian reoviruses (ARV) [9-11]. According to genome alignment of GCRV with its homologous proteins, except for the similarity presented in structural proteins between Genus *Aqureovirus* and *Orthoreovirus*, some nonstructural proteins also remain conserved domain that performed similar function in virus replication cycle, indicating that both Genus *Aqureovirus* and *Orthoreovirus* share common molecular morphogenesis during virus infection [9,10]. Notablely, uNS(or uNSC), a MRV nonstructural protein, has been demonstrated that it is sufficient for forming phase-dense viroplasmic inclusion bodies (VIB) in the cytoplasm of transiently transfected cells [11-17]. The VIB like structures formed by single uNS are similar in its appearance to globular inclusions formed in MRV infected cell, suggesting that uNS is able to form matrix of viral factories [17]. Different from the dominated role played by μNS in VIB formation in MRV, another nonstructural protein σNS is also recognized to be related to form VIB like structures by interaction with μNS [18-20]. Besides, the core protein μ2 and σ2 were also verified to play very important roles by interacting with μNS in the formation of VIB. Recent progress indicated that the core protein μ2, known as a cellular microtubule associated protein, is recognized to determine the morphology of VIB (showed either globular or filamentous) in MRV [17,21]. Studies on reassortment suggest that μ2 determines viral strain differences in transcriptional efficiencies of core particles [22,23], and also displays both ssRNA and dsRNA binding abilities, which demonstrates that μ2 possesses both nucleoside triphosphatase (NTPase) and RNA-triphosphatase (RTPase) activities [24-26]. As a homologue of μ2 protein, the VP4 protein in GCRV was presumed to have a similar function to μ2 of MRV.

In light of the previous investigation on GCRV genome and molecular biology characterization [9,10,27-29], nonstructural protein NS80 of GCRV, the analog of μNS protein in MRV, has been previously identified to be related to viral inclusion formation during virus replication and particle assembly [28]. The protein VP4 was predicted to be a core protein possessing NTPase activity played in viral genome transcription, and identified to have interaction with NS80 [30]. To characterize the core protein VP4, the recombinant plasmids coding full length of VP4 gene were constructed and expressed in both prokaryotic and mammalian cells in this study. The recombinant His-tag fusion VP4 product expressed in E.*coli* was identified by Western blotting with both His-tag specific monoclonal antibody and GCRV polyclonal antibody. Besides, the expression of VP4 in GCRV infected CIK cells can be detected by Immunofluorescence (IF) using prepared anti-VP4 polyclonal antibody, which appeared in granules structure concentrated mainly in the cytoplasm. To confirm the VP4 to be a structural component in viral particle, both GCRV core and infected cell lysate were identified by Immunoblotting (IB) assay using VP4 polyclonal antibody. In particular, the VP4 protein was observed in the cytoplasm and nucleus with diffused distribution in transfected cells, suggesting that VP4 protein may play specific mechanism in GCRV replication. The present study will lay a foundation for further investigating the precise function of VP4 and the association with other proteins.

## Methods

### Virus and cell lines

CIK (*Ctenopharyngodon idellus* kidney) cell line and Vero cell line were prepared for the GCRV infection and transfection assays as described previously [27,28]. The cells were grown in Eagle’s minimum essential medium (MEM, Invitrogen,USA) and Dulbecco’s Modification of Eagle’s Medium (DMEM, Invitrogen, USA) containing 2 mM L-glutamine supplemented with 10% of fetal bovine serum respectively. GCRV-873, the original isolate of GCRV proliferated in CIK monolayer cells as described previously [31,32], was used in this study.

### Construction of recombinant plasmids

To construct the recombinant plasmid for expression in prokaryotic system, the primers targeting GCRV S5 contained specific restriction sites were designed based on Genbank sequence (AF403391). The sense primer was: CATCTGCAGATGATCACCATTGTGGTT (*Pst*I site underlined); the anti-sense primer was: GCT‒AAGCTT GTAGCGTCAGGACTCCTC (*Hind* III site underlined). RT-PCR was performed by using the purified whole GCRV genome as template to amplify GCRV S5 segment as described previously [28]. The S5 PCR product was purified, and was then ligated into pRSET-C vector (Invitrogen,USA) which had been linearized with *Pst* I and *Hind* III (TaKaRa, Dalian, China). The constructed recombinant, named as pR/VP4, was transformed into *E. coli* and verified by both restriction enzyme digestion and PCR amplification as described elsewhere [28]. The confirmed positive recombinant plasmids were also sequenced by Invitrogen Biotechnology Co., Ltd (Shanghai, China).

To express VP4 protein in mammalian cells, pcDNA/VP4 and pEGFP/VP4 recombinants were generated. A primer pair containing forward primer (GCRV-S5-SN): GCTAAGCTTGCCAGGATGATCACCATTG (*Hind* III site underlined), reverse primer (GCRV-S5-AS): TAGAGATCTTCAAACCCCGGTCGAGGT (*Bgl* II site underlined) was designed for genome amplification of GCRV full-length S5 respectively. Furthermore, a primer pair containing forward primer:CGC‒AAGCTTACATGATCACCATTGTGGTT (*Hind* III site underlined),reverse primer: TAGAGATCTTCAAACCCCGGTCGAGGT(*Bgl* II site underlined)was designed to generate a fusion of full length S5 to C terminus of GFP. The amplified fragments were cloned into pcDNA3.1+ (Invitrogen,USA) or pEGFP-C1 (Clontech,USA) vector which had been cut with *Hind* III and BamH I (the isocaudamer of *Bgl* II) and named as pcDNA/VP4 and pEGFP/VP4 respectively. The positive recombinants were double identified before performing final sequencing as described above.

### Expression of recombinant protein VP4 and antisera praparation

In order to express VP4 in E. *coli*, the positive recombinant transformant was grown in SOB medium shaking at 37°C and then induced by 1 mmol/L IPTG until the cell reached logarithmic growth phase, meanwhile, the pRSET-C plasmid was supplied at the same as control. After being induced for 1 h, 3 h, 5 h at 28°C, all the lysate extracts of expressed bacteria were resuspended in phosphate-buffered saline (PBS), and stored at −30°C for further analysis. The purification of His-tag fused VP4 protein was performed according to the ProBond^TM^ Resin kit instruction (Invitrogen,USA).

To raise antibodies against recombinant VP4 protein, BALB/C mice were immunized with 100 ul (~4 ug/ul) of purified VP4 protein in equal volume of complete Freund's adjuvant and boosted three times with the same amount of antigens in incomplete Freund's adjuvant at 2 weeks intervals. Ten days after the final infection, the mice were bled, and serum was separated, and stored at-80°C. The antibody titer was determined by ELISA according to report elsewhere [33].

### SDS-PAGE and immunoblotting analysis

All samples were mixed 1:1 with 2 × protein loading buffer (20 mM Tris–HCl [pH 8.0], 2% SDS, 100 mM DTT, 20% Glycerol, 0.016%Bromophenol Blue), disrupted by boiling for 5 min, and separated in 10% SDS-PAGE by electrophoresis. Staining was performed with Coomassie brilliant blue R-250 (Sigma, USA). For immunoblotting, proteins in the gel were transferred to a PVDF (polyvinylidene Fluoride) membrane by Semi-dry transfer cell following the instrument’s instruction (Bio-Rad). After being blocked with 3% bovine serum albumin (BSA), mouse anti-His-tag, mouse anti-VP4, or rabbit anti-GCRV andibody was used as the primary antibody to detect collected protein samples. The secondary antibody was alkaline phosphatase-conjugated goat anti-mouse immunoglobulin G (IgG) or goat anti-rabbit IgG. All the samples were observed by developing with AP substrate solution (NBT/BCIP).

### Infection and purification of virus core

Confluent monolayers of CIK cells were infected with GCRV at a multiplicity of infection (MOI) of 2–10 PFU/cell. Following 1 h of adsorption, cells were washed with PBS to remove the inoculums and fresh medium supplemented with 2% of fetal bovine serum was added at 28°C for viral propagation. The virus-infected CIK cells could be fixed for IF assay until the cytopathic effects (CPE) were observed. At the same time, the rest virus infected cell supernatants were collected and stored at −30°C for viral particle purification. The intact virion and core were isolated by CsCl density gradient centrifugation as described elsewhere [27].

### Transfection and IF microscopy

After confirming the correctness of recombinants, the identified recombinant pcDNA/VP4, and pEGFP/VP4 plasmids were used to transfect Vero cells respectively based on transfection kit user introduction (Lipofectamine 2000, Invitrogen,USA) or following previous report as described elsewhere [28]. pcDNA3.1+ and pEGFP-C1 were also used to transfect Vero cells as the negative control.

Infected or transfected cells were fixed for 20 min at room temperature(RT) with 2% paraformaldehyde in PBS, and then permeabilized with 0.1% TritonX-100 for 5 min. Permeabilized cells were blocked with PBS containing 3% bovine serum albumin (PBS-BSA) for 1 h at 37°C prior to incubation with primary VP4 specific antibody diluted in PBS-BSA. After binding with first antibody, cells were washed three times with PBS, and then incubated with secondary fluorescein isothiocyanate(FITC)-tagged goat anti-mouse antibody diluted in PBS-BSA for 1 h at 37°C. Hoechst staining was applied for the localization of the cell nucleus, and the tested samples were observed using Olympus-IX51 inverted microscope equipped with phase and fluorescence optics. Collected images were processed with Pro-Eexpress 6.3 (Olympus) and Photoshop (Adobe Systems).

## Results

### Identification of expressed VP4 in E. *coli*

**Figure 1:**
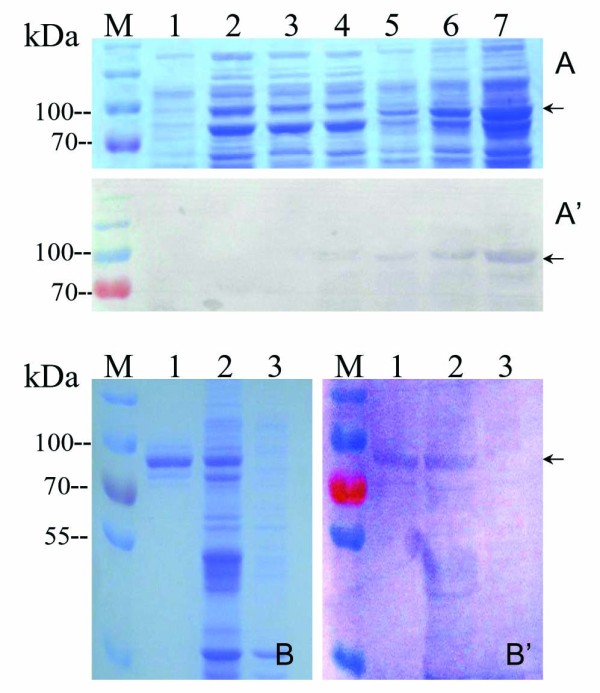
**Identification and purification of expressed VP4*.*A**: SDS-PAGE analysis of induced recombinant VP4 expression*.* M, Standard protein marker; Lane1, induced empty vector expression for 3 h as control; 2–4, cell supernatant of induced VP4 expression for 1,3,5 h respectively; 5–7, cell pellet of induced VP4 expression for 1,3,5 h respectively; **A**^**’**^: Western blot analysis of expressed protein samples corresponding to lane 1–7 in **A** with mouse anti His-tag antibody;B: SDS-PAGE analysis of purified VP4 protein. M, Standard protein marker; Lane 1, purified VP4 protein; 2, expressed VP4 protein in cell lysate pellet ; 3, empty vector control ; B’: Western blot analysis of purified VP4 protein matching Lane 1–3 in B with rabbit anti-GCRV antibody.

To obtain the recombinant VP4 fusion protein, the constructed plasmid pR/VP4 was transformed into BL21(DE3)plysS cells and induced by using 1 mmol/L IPTG at 28°C for 1, 3, 5 h respectively. As shown in Figure 1A and A’, the full length of VP4 was successfully expressed in E. *coli,* which was around 83 kDa consistent with its predicted value because the N-terminal tag increased the size of expressed VP4 recombinant fusion protein by approximately 3 kDa. In addition, the product of the VP4 fusion protein was concentrated in the pellet rather than supernatant of the cell lysate, indicating that the induced VP4 fusion protein expression was presented in the form of inclusion body. The validity of expressed fusion protein was further confirmed by Western blot using a His-tag monoclonal antibody. The result showed the expressed fusion protein was able to bind to the His-tag antibody specifically, suggesting that the recombinant protein was the His-tag fusion VP4 protein as expected.

Following VP4 expression, the purification of the His-tag fusion protein was performed with a Ni^2+^-Chelating resin column. A nearly single target band corresponding to the correct molecular weight of the interest protein was detected, indicating that the target protein could be conjugated to the resin. Furthermore, the purified protein and cell lysate could cross immunologically with GCRV polyclonal antibody (Figure 1B and 1B’), demonstrating that the recombinant fusion VP4 protein is GCRV related antigen that belonged to viral structural protein, which could be further used as an immunogen for VP4 antibody production in animal.

### Detection of expressed VP4 in GCRV infected cells

**Figure 2:**
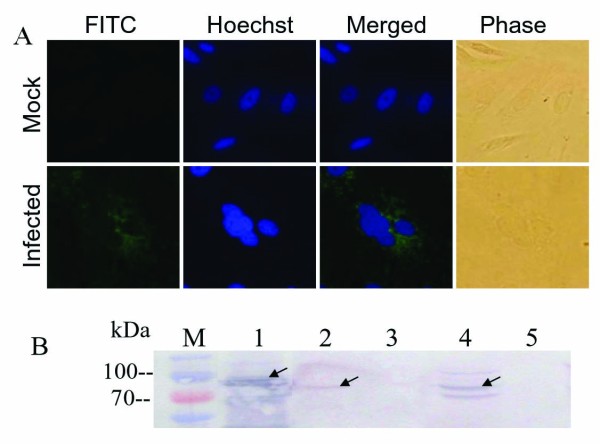
**Indirect IF and IB assays of the VP4 protein. A:** Indirect IF assay of the VP4 protein in GCRV infected CIK cells. Cells were probed with the VP4 antiserum and labeled with FITC-conjugated goat anti-mouse immunoglobulin G(IgG), counterstained with hoechst to visualize the nuclei, Mock infection (upper panel); GCRV infected CIK cell (lower panel). **B:** IB assay of the VP4 protein in GCRV and its infected CIK cells using mouse anti-VP4 polyclonal serum as the primary antibody followed by goat anti-mouse IgG coupled to alkaline-phosphatase. M, Standard protein marker; Lane1, Recombinant His-tag fusion VP4 protein; 2–3, purified GCRV core and top component particles; 4–5, GCRV-infected and mock-infected CIK cell lysate.

In contrast with nonfosogenic MRV, fosogenic GCRV can produce a typical cytopathic effect (CPE) with large multinucleated syncytia in its sensitive cells. To investigate whether VP4 protein is involved in GCRV replication, the expression of VP4 protein was detected in GCRV infected cells by utilizing indirect IF and IB assays. The result of IF examination showed that VP4 protein was dispersed in cytoplasm of GCRV infected cells along with accumulating of nucleus, there is no fluorescence presented in normal CIK (or mock infected) cells, indicating that expressed VP4 can be detected in virus infected cells. To further verify VP4 protein expression, IB was also conducted by using the virus samples from purified virus particles including core and top component as well as infected cell lysate. The result, as shown in Figure 2B, indicated that the VP4 protein was expressed in GCRV infected CIK cells, and could be detected in viral core, but not a constitution of the outer shell.

### Subcellular localization of VP4 in transfected cells

**Figure 3:**
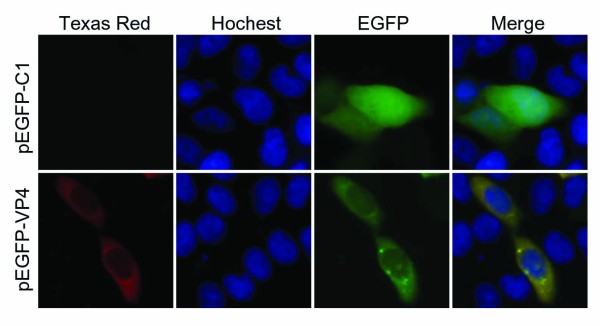
**Indirect IF assay of the EGFP-VP4 protein in transfected cells.** Vero cells were transfected with pEGFP/C1(upper panel) or pEGFP/VP4(lower panel) for 24 h and then subjected to the indirect IF assay with the VP4 antiserum, labeled with Texas Red-conjugated goat anti-mouse IgG, counterstained with hoechst to visualize the nuclei. The pEGFP-C1 was used as negative control.

**Figure 4:**
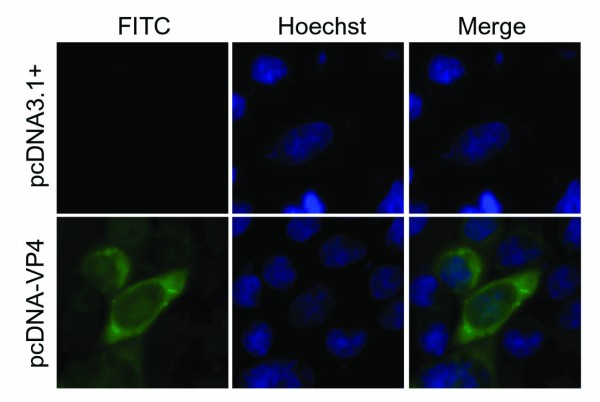
**Subcellular localization of the VP4 protein in transfected cells.** Vero cells were transfected with pcDNA/VP4 (lower panel) or pcDNA3.1(+) (upper panel) for 24 h and then subjected to the indirect immunofluorescent assay with the VP4 antiserum, labeled with FITC-conjugated goat anti-mouse IgG, counterstained with hoechst to visualize the nuclei. The pcDNA3.1(+) was used as negative control.

To investigate subcelluar location of VP4 protein, the recombinant VP4 plasmids pEGFP/VP4 and pcDNA/VP4 were transfected into Vero cells respectively. IF examinations showed (Figures 3 and 4) that the EGFP-fused VP4 protein expression in transfected cells was diffusely distributed in all the cells including cytoplasm and nucleus, and the phenotype was confirmed by non-fused VP4 expression in pcDNA/VP4 transfected cells. In addition, it appeared that the distribution of VP4 mainly concentrated in cytoplasm with few granules at the margins of the nucleus in transfected cells. Based on this evidence, we hypothesized as part of the current study that VP4 not only plays a major role related to VIB structure and morphogenesis in cytoplasm, but also may function in nucleus during viral genome transcription.

## Discussion

Reovirus replication and assembly are thought to occur within viral inclusions that form in the cytoplasm of infected cells [12-15]. It has been clarified that viral inclusions contain viral proteins, both complete and incomplete viral particles, and dsRNA, where are thought to be the site of viral RNA replication and packaging into progeny particles [12,13,17]. The nonstructural proteins μNS and σNS and minor core protein μ2 are identified collectively to be required for the genesis and maturation of viral inclusions in MRV infected cells [17,18]. Genome sequence analysis indicates that VP4 protein, encoded by GCRV S5 segment, is comprised of 728 amino acids with approximately 80 kDa in its size. The protein was predicted to be a core protein in view of a strong similarity (about 21%) with μ2 in MRV. Furthermore, the two motifs spanning from aa (amino acid) residues of 406–421 and 437–446 corresponding to the NTPase activity, were also conserved by sequence alignment with MRV μ2 protein. Based on the analysis above, VP4 protein was predicted to function as part of cofactors of VP2 protein to be active in viral genome transcription as well as its important roles in VIB structure maturation in reovirus replication pathway.

Different from nonfusogenic mammalian reovirus, GCRV can produce a typical cytopathic effect by promoting of large multinucleated syncytia in its sensitive cells, indicating it is a fusogenic reovirus. Previous report indicated that single nonstructural protein NS80 can form globular inclusion-like structures in its transfected cells [28], and the interaction between NS80 and VP4 was also identified by yeast two-hybrid (Y2H) system [30]. The result of IF examination showed in this study indicated that VP4 protein was dispersed in cytoplasm of GCRV infected cells with a small aggregates, which was suspected to be sites of active viral genome replication and core assembly. To further verify the VP4 protein expression and assembly, IB was conducted by using the samples from both purified viral particles and infected cell lysate. The result showed (Figure 2B) that the VP4 protein was presented in low copy number in the virion but expressed comparatively abundant in infected cells, suggesting some of the VP4 might associate with microtubules and contribute to formation of viral factories that is similar to μ2 protein in MRV [14,17,21]. Interestingly, despite the exclusively cytoplasmic replication strategy of reovirus, the VP4 protein expressions in pEGFP/VP4 and pcDNA/VP4 transfected Vero cells were both observed to be diffusely distributed in cytoplasm and nucleus, suggesting that VP4 protein may play some role in the nucleus by regulating cell cycle besides its predicted cytoplasmic function in GCRV replication. Further investigation may need to be performed to reveal the nucleus related specific mechanism of VP4 protein in viral infection.

In this study, the recombinents of VP4 were constructed and expressed in both prokaryotic and mammalian cells. The polyclonal VP4 antibody was generated and confirmed specific to its antigen. Furthermore, the expressions of VP4 in GCRV infected CIK cells, detected by IF or IB using prepared VP4 polyclonal antibody, were showed in granules structure concentrated mainly in the cytoplasm, suggesting that the core protein component VP4 might be expressed and assembled in the cytoplasm in VIB. It may need to note that the VP4 protein was observed in the cytoplasm and nucleus with diffused distribution in transfected cells, which suggest that VP4 protein may play part of role in nucleus and have a specific mechanism in GCRV pathogenesis.

## Conclusion

Our results provided in this study indicated that the VP4 is a component of GCRV core. The cellular localization of expressed single VP4 is correlated with the predicted function. Taking together with our previous study that VP4 have interaction with NS80, further functional identification of VP4 will provide a useful information to reveal its role in VIB formation and GCRV replication cycle.

## Competing interests

The authors declare that they have no competing interests.

## Authors’ contributions

QF designed the experiments. LMY, HG carried out the experiments. XYS and LS participated in VP4 protein purification and antibody preparation. QF, LMY and HG analyzed the data and wrote the paper. All authors read and approved the final manuscript.
